# Underwater closure of a sigmoid perforation with through-the-scope twin clip

**DOI:** 10.1055/a-2738-7090

**Published:** 2025-11-21

**Authors:** Haitao Shi, Xiaosa Jiang, Xiangyue Qi, Tianqi Xu, Ning Xie, Lu Li, Bin Qin

**Affiliations:** 1 117799 Department of Gastroenterology, Xiʼan Jiaotong University Second Affiliated Hospital, Xiʼan, China

Perforation represents a serious complication of colonoscopy. Endoscopic closure is often challenging due to high tension at the site of full-thickness defects. A 67-year-old man underwent surveillance colonoscopy following right hemicolectomy for colorectal cancer. During the procedure, he developed acute left abdominal pain with localized rigidity. The endoscopist withdrew the endoscope for further evaluation, revealing a perforation in the sigmoid colon, 20 cm from the anal verge. We promptly exchanged the colonoscope for a gastroscope with a transparent cap. Upon re-examination, a perforation is found in the sigmoid colon. It is approximately 1 cm in diameter, and yellow omental tissue is observed ( Fig. 1 **a** ). To reduce air-induced tension and improve visualization, air was suctioned and the site was submerged in saline. Saline immersion resulted in reduction of the wound size and gathering of the surrounding mucosa ( Fig. 1 **b** ). A reopenable through-the-scope twin clip (TTS-TC) was selected for its superior grasping force and longer arms. After clamping the intestinal wall of the perforation on the anal side, the wound was pulled close to the oral side and was clamped using the clip on the other side of the TTS-TC ( Video 1 ). The clips on both sides of the TTS-TC were released and the wound was successfully closed ( Fig. 1 **c** ). Conventional clips were then deployed to reinforce the closure ( Fig. 1 **d** ).

**Fig. 1:**
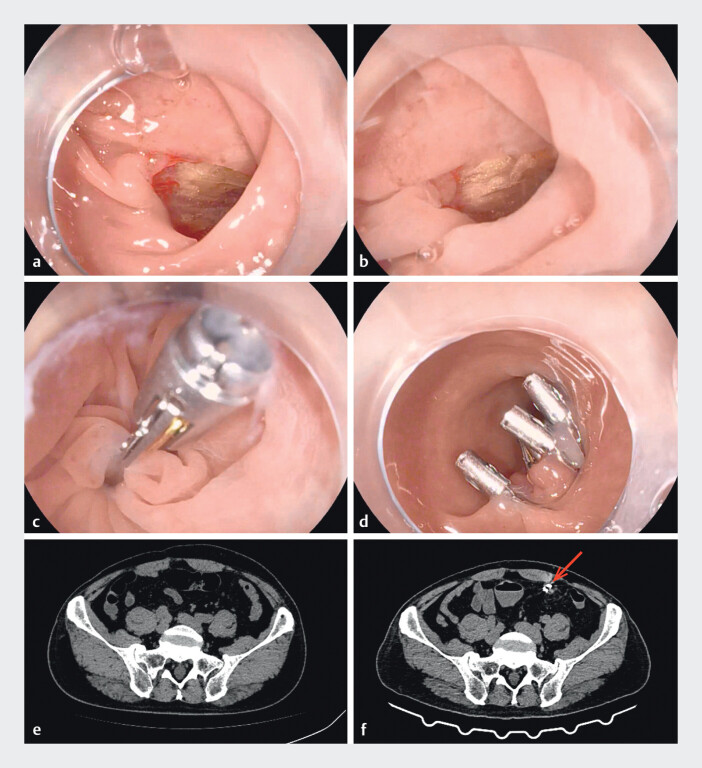
Images of a sigmoid perforation closed underwater using a through-the-scope twin clip. **a** Perforation observed in a conventional endoscopic view. **b** Underwater view of the perforation. **c** Closure using a reopenable through-the-scope twin clip (TTS-TC). **d** The perforation after successful wound closure. **e** Abdominal CT image obtained prior to surgery. **f** Abdominal CT image obtained 48 hours after surgery. The red arrow indicates the metallic clips and minimal exudation.

A clinical case of underwater closure of a sigmoid colon perforation performed with a through-the-scope twin clip.Video 1

Postoperatively, the patient was managed with 48 hours of fasting and prophylactic antibiotics. His abdominal pain quickly resolved without fever or leukocytosis. Compared with the preoperative abdominal CT image ( Fig. 1 **e** ), the abdominal computed tomography (CT) at 48 hours postoperatively ( Fig. 1 **f** ) demonstrated metallic clips and minimal exudation, with no evidence of pneumoperitoneum.

The sigmoid colon is the most frequent site of colonoscopy-related perforation. Sharp angulation and high tissue tension often complicate endoscopic repair. The underwater procedure facilitates closure of perforation by reducing the wound size and tension, simplifying the clip application, and potentially mitigating gas-related complications.

Endoscopy_UCTN_Code_CPL_1AJ_2AJ

## Notes

Endoscopy E-Videos https://eref.thieme.de/e-videos
*E-Videos* is an open access online section of the journal *Endoscopy* , reporting on interesting cases and new techniques in gastroenterological endoscopy. All papers include a high-quality video and are published with a Creative Commons CC-BY license. Endoscopy E-Videos qualify for HINARI discounts and waivers and eligibility is automatically checked during the submission process. We grant 100% waivers to articles whose corresponding authors are based in Group A countries and 50% waivers to those who are based in Group B countries as classified by Research4Life (see: https://www.research4life.org/access/eligibility/ ). This section has its own submission website at https://mc.manuscriptcentral.com/e-videos .

